# In Vitro Gastrointestinal Stability and Functional Evaluation of Yogurt Fortified With Paraprobiotics and Postbiotics

**DOI:** 10.1002/fsn3.71787

**Published:** 2026-04-15

**Authors:** Gökçe Eminoğlu

**Affiliations:** ^1^ Department of Dairy Technology Ankara University Faculty of Agriculture Ankara Turkey

**Keywords:** antioxidant activity, in vitro digestion, paraprobiotics, phenolic compounds, postbiotics, probiotic yogurt

## Abstract

In this study, yogurt formulations with paraprobiotic (heat inactivated *Lactobacillus acidophilus* ) and postbiotic (autolyzed and dried *Saccharomyces cerevisiae* ) additions were developed to improve the techno‐functional properties of yogurt/probiotic yogurts. Yogurt/probiotic yogurt samples were prepared by adding 0.5% autolyzed and dried *S. cerevisiae* and 10^9^ cfu/mL heat‐killed *L. acidophilus* cells and stored at 4°C ± 1°C for 21 days. Using a standard in vitro gastrointestinal digestion model simulating digestion in the gastric and intestinal phases, bacterial survival, antioxidant and phenolic compounds, and antimicrobial properties in yogurt samples were determined and compared with the pre‐digestion. The addition of inactive *L. acidophilus* cells had no significant effect on the physicochemical, microbiological, antioxidant or antimicrobial properties of yogurt samples. In contrast, the physical, chemical, and sensory properties of the samples containing autolyzed and dried *S. cerevisiae* were affected, and these effects also varied with storage time. The antioxidant and phenolic contents of the samples increased by 69%–117% and 8%–25%, respectively, during the gastric digestion compared to pre‐digestion levels, whereas after intestinal digestion, these values reached 122%–208% and 135%–172%. In vitro digestion analyses revealed that samples containing postbiotics and paraprobiotics exhibited higher levels of bioavailable compounds throughout the digestion stages. These findings suggest that the addition of postbiotics and paraprobiotics may be an effective strategy to improve the functional properties of fermented dairy products.

## Graphical abstract

The study findings revealed that the antioxidant and phenolic contents of the samples increased by 69%–117% and 8%–25%, respectively, during gastric digestion compared to pre‐digestion, and these values reached 122%–208% and 135%–172% after intestinal digestion. The bioaccessibility of antioxidant and phenolic compounds of probiotic yogurts containing autolyzed *Saccharomyces cerevisiae* and heat‐inactivated *Lactobacillus acidophilus* cells after in vitro digestion was higher than that of plain yogurts.

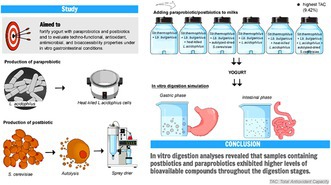

## 1 Introduction

Globally, consumers are experiencing rapid changes in their food consumption habits. Healthier, safer, minimally processed, and nature‐identical foods are now more appealing. Although this trend of consumers actually dates back to old times, this process has accelerated, especially with the Covid‐19 pandemic (Taşkoparan et al. 2025). Functional foods are any food or food ingredients that provide health benefits that go beyond basic nutrition (Alizadeh Behbahani et al. 2024). Functional foods are foods fortified with components including vitamins, proteins, fibers, probiotics, and prebiotics that confer health advantages to humans. The most often employed functional ingredients in next generation food formulations include probiotics, prebiotics, postbiotics, phytochemicals, microalgae, polyunsaturated fatty acids, and sulfur‐containing components (Alu'datt et al. 2024).

Yogurt is a fermented dairy product produced by lactic acid fermentation provided by symbiotic yogurt cultures. In addition to being considered a product with health benefits, yogurt is also a suitable carrier for probiotic microorganisms (Akan 2022). The increasing demand for health‐enhancing foods is leading the dairy industry to produce functional probiotic yogurts to meet this need (Olson and Aryana 2022). Probiotic bacteria are live microorganisms that provide health benefits to the host when consumed in adequate amounts (Echresh et al. 2024). Yogurt is considered the most common medium for the administration of probiotic bacterial strains that are generally considered safe for consumers. In recent years, public interest in fermented goods containing probiotic bacteria has grown due to increasing evidence of the benefits of probiotics for the management of gut microbiota and antimicrobial activity (Mehdizadeh et al. 2021).

To provide health benefits, probiotics must survive in sufficient numbers in the product until they reach the intestines. To show these effects, the minimum viable probiotics in the final product must be 10^6^–10^7^ cfu/g or mL at the time of consumption (Mehdizadeh et al. 2019). However, recent studies have shown that non‐viable microorganisms or their metabolites can also be biologically active in the host (Aguilar‐Toalá et al. 2018; Taverniti and Guglielmetti 2011). Therefore, there has been an increasing number of studies emphasizing the beneficial effects of non‐viable cells and their metabolites, referred to as “paraprobiotics” and “postbiotics.”

Paraprobiotics, the non‐living versions of probiotics, are characterized as nonliving bacteria that support human health. Paraprobiotics are obtained from inactivated organisms after chemical or mechanical stress. Cells undergo mechanical/chemical damage due to damage to the cell membrane or degradation of DNA strands (Siciliano et al. 2021). After inactivation, cell viability is checked using plates in appropriate culture media. The use of paraprobiotics provides several advantages such as no risk of passage from the intestinal lumen to the blood, stability against the transfer of antibiotic resistance genes, and limited interference with the normal colonization of the intestinal microbiota (Nataraj et al. 2020).

Postbiotics refer to metabolites or soluble compounds with biological benefits produced by probiotic bacteria or other food‐grade microorganisms (Aguilar‐Toalá et al. 2018). Postbiotics include metabolic byproducts of probiotic strains including secreted enzymes, proteins, short‐chain fatty acids, vitamins, and phenols. Postbiotics are notable for their long shelf life, safety dose parameters, and the inclusion of various signaling molecules with antioxidant, anti‐inflammatory, antihypertensive, anti‐obesogenic, immunomodulatory, antiproliferative, and hypocholesterolemic activities (Aguilar‐Toalá et al. 2018; Gurunathan et al. 2024; Park et al. 2022; Pimentel et al. 2021, 2023; Tain et al. 2024). Although the mechanisms of the health effects of postbiotics have not been fully elucidated, they suggest that they may contribute to host health by improving certain physiological functions.

Including paraprobiotics and postbiotics in the diet may provide greater benefits than probiotics due to their lesser interaction with food components. Similar to probiotics, they provide various health benefits when taken in adequate amounts. Consequently, since they do not need to remain viable throughout shelf life, they can be included before heat treatment, facilitating the production, transportation and storage of products (Aggarwal et al. 2022; Piqué et al. 2019).

Considering the above information, the use of paraprobiotics and postbiotics may be a worthwhile alternative to produce functional yogurt compared to probiotics. Innovative and healthier advancement of yogurt, distinguished among probiotic dairy goods, possesses the capacity to enhance both food science and technology as well as consumer wellness. One of the methods to be utilized for this objective is the acquisition of products fortified with postbiotics and paraprobiotics, which are the focus of this research. Numerous research have examined the production, characterization, and possible use of paraprobiotics and postbiotics in dairy products (Aggarwal et al. 2022; Barros et al. 2020; Cuevas‐González et al. 2020; Molaee Parvarei, Fazeli, Mortazavian, Sarem Nezhad, Mortazavi, et al. 2021). Recent studies have shown that postbiotics added to yogurt have antibacterial, antioxidant, and cholesterol‐lowering effects (Davarzani et al. 2024). In addition, it has been reported that the addition of postbiotics to yogurt increases antioxidant capacity (Pham et al. 2024). In some studies, it has been noted that the addition of paraprobiotics to yogurt increases the viscosity and water holding capacity of yogurt and positively affects its sensory properties (Molaee Parvarei, Fazeli, Mortazavian, Sarem Nezhad, and Mortazavi 2021; Molaee Parvarei, Fazeli, Mortazavian, Sarem Nezhad, Mortazavi, et al. 2021; Pham et al. 2024). However, there is no comparative study in the literature on paraprobiotics and postbiotics in yogurt and probiotic yogurts. This study aims to fortify yogurt, a functional dairy product, with postbiotics and paraprobiotics using an innovative approach to enhance its health benefits, achieve a unique composition, and assess the bioaccessibility of bioactive molecules in samples obtained after in vitro digestion simulation.

## 2 Materials and Methods

### 2.1 Chemicals

Culture media, Folin–Ciocalteu reagent, sodium carbonate, gallic acid, and ethanol were obtained from Merck (Darmstadt, Germany). 2‐Diphenyl‐1‐picrylhydrazyl (DPPH) was obtained from TCI Chemicals India. All chemicals and reagents used in this study were of analytical or HPLC grade. Pepsin (P7000 from porcine gastric mucosa), pancreatin (P1750 from porcine pancreas), and bile enzymes (P8631) were obtained from Sigma‐Aldrich (Steinheim, Germany). Paper disks used in the disk diffusion technique were obtained from Bioanalyse (Ankara, Türkiye).

### 2.2 Production of Autolyzed and Dried *S. cerevisiae*

*Saccharomyces cerevisiae* (NCYC‐R‐625) was obtained by spray drying after being grown in a molasses‐based medium, supplied by a commercial company (Pakmaya, İzmit, Türkiye). Instant yeast preparation *S. cerevisiae* NCYC‐R‐625 was produced using distilled water at a concentration of 30.0 g per 100 mL. Autolyzed yeast was obtained by slightly modifying the method described by Takalloo et al. (2020). The solution was subsequently heated to 80°C. The temperature was sustained for 2 h with gentle stirring. This was succeeded by drying with a spray dryer. The spray drying process was conducted using a laboratory‐scale spray drier (B290; Buchi, Flawil, Switzerland). An autolyzed *S. cerevisiae* NCYC‐R‐625 solution (15.0 g/100 mL) was produced for drying. The drying conditions consisted of an atomization pressure of 65 mbar, a flow rate of 1.40 mL min^−1^, and a gas pressure of 6 bar. The inlet and outlet temperatures of the spray dryer were 180°C and 100°C, respectively. Dried samples were preserved in securely sealed opaque containers at 20.0°C ± 2.0°C. The dry matter, protein, glucan‐mannan, ash, and fat contents of powdered yeast cells were 94.5% ± 2.5%, 40%, 33%, 6%, and 1%, respectively.

### 2.3 Preparation of Heat‐Killed Cells

Paraprobiotics were obtained using *L. acidophilus* La‐5 in lyophilized powder form. To prepare heat‐inactivated probiotic cells, the bacteria were suspended in distilled water at a concentration of 10^7^ cfu/mL and then autoclaved at 105°C for 15 min. Bacterial viability was assessed immediately after autoclaving, and no colonies were found on the plates, confirming complete inactivation (Molaee Parvarei, Fazeli, Mortazavian, Sarem Nezhad, Mortazavi, et al. 2021).

### 2.4 Preparation of Yogurt Samples

**FIGURE 1:**
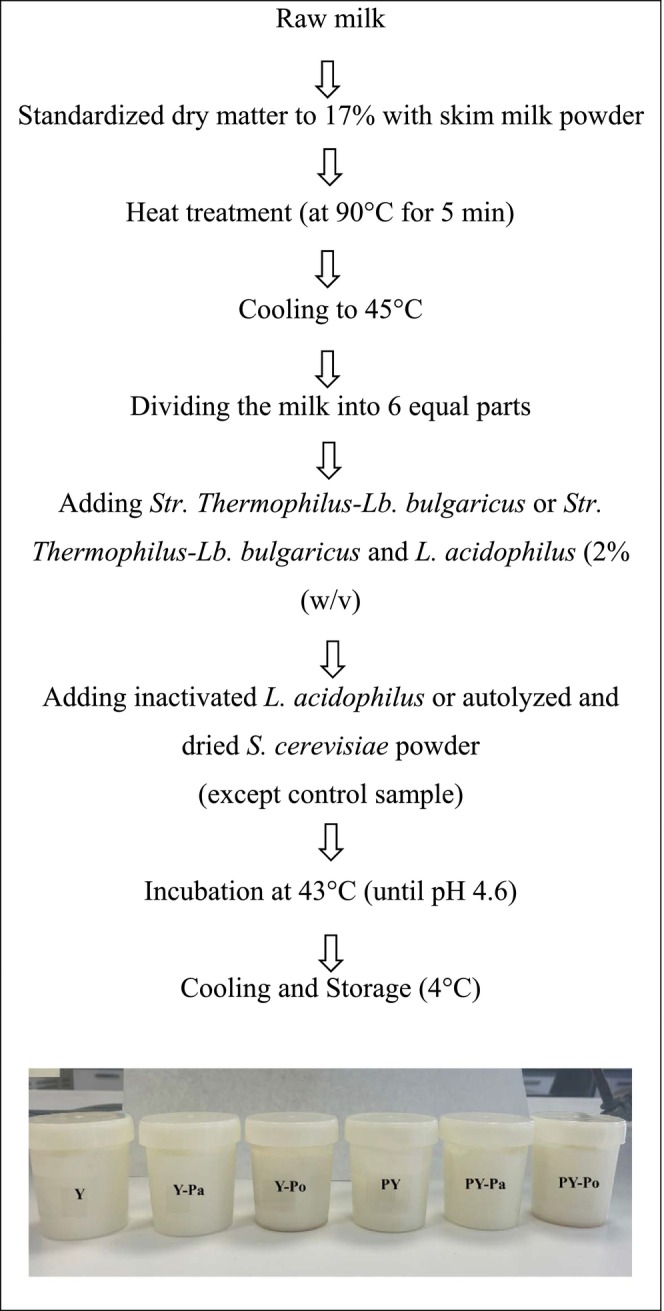
Flow diagram of yogurt production.

Raw milk (12.3 ± 0.2 dry matter, 3.5% ± 0.3% protein, 3.6% ± 0.1% fat, and pH 6.7 ± 0.0) required to prepare yogurt samples was provided by Ankara University, Faculty of Agriculture, Pilot Dairy Plant. The dry matter content of raw milk was standardized to 17% with skimmed milk powder. Then, milk was pasteurized at 95°C for 5 min and then cooled to 43°C–45°C. After the milk was cooled, commercial yogurt starter culture or probiotic culture ( *L. acidophilus* ) and heat killed *L. acidophilus* or autolyzed *S. cerevisiae* were added and then incubated. Fermentation was terminated when the pH reached 4.6. After incubation, yogurt samples were taken to 4°C. The experimental design of the study, which involved a total of six different samples, and the yogurt production flow diagram are presented in Table 1 and Figure 1, respectively.

**TABLE 1:** Codes of the samples and experimental design of the study.

Sample code	Treatments/formulation
Y	Non‐fortified yogurt (control)
Y‐Pa	Yogurt fortified with 10^7^ log cfu/mL heat‐killed *Lactobacillus acidophilus* cell
Y‐Po	Yogurt fortified with 0.5% autolyzed and dried *Saccharomyces cerevisiae*
PY	Non‐fortified probiotic yogurt
PY‐Pa	Probiotic yogurt fortified with 10^7^ log cfu/mL heat‐killed *L. acidophilus* cell
PY‐Po	Probiotic yogurt fortified with 0.5% autolyzed and dried *S. cerevisiae*

### 2.5 Gross Composition of Yogurt Samples

The gross composition of the yogurt samples was determined by analyzing dry matter, fat, and protein contents. Fat was measured using the Gerber method, dry matter through gravimetric analysis, and protein by the Kjeldahl method (AOAC 1995). A combined electrode pH meter (Cyberscan pH 300; Eutech Instruments) was utilized to measure the pH value of the samples.

### 2.6 Enumeration of Bacteria

Cell populations of *S. thermophilus* , *Lb. bulgaricus*, and *L. acidophilus* were determined using the pour‐plate technique as described by Donkor et al. (2007) with slight modifications. Ten grams of yogurt samples were diluted with 90 mL of Ringer's solution. Samples were then homogenized using a laboratory mixer (Bag Mixer 400 VW; Interscience, France) for 2 min, followed by serial dilutions prepared. For counting *S. thermophilus* , and *Lb. bulgaricus*, the petri dishes were incubated in M17 and MRS agar at 37°C for 48 h, respectively. To determine *L. acidophilus* counts, the plates were incubated in MRS‐Sorbitol agar at 37°C for 72 h.

### 2.7 In Vitro Digestion

Gastric and intestinal digestion of yogurt samples was performed according to the INFOGEST method (Brodkorb et al. 2019). The components of gastric and intestinal fluids were calculated using the INFOGEST method.

To simulate gastric digestion, 10 g of yogurt sample, 8 mL of gastric juice, 0.5 mL of pepsin (2000 U/mL) solution, and 5 μL of calcium chloride (0.3 M) were added and adjusted to pH 2.5–3 with 1 M HCl. Next, distilled water was added to bring the total volume of the mixture to 20 mL, and it was mixed in a shaker incubator (MCİ55; Microtest, Turkey) at 37°C for 2 h. After gastric digestion was completed, 10 mL aliquots were collected from each sample.

To simulate intestinal digestion, 10 mL of the gastric digest was mixed with 4.25 mL of intestinal juice, 2.5 mL of pancreatin (100 U/mL), and 1.25 mL of bile (10 mM). The pH of the mixture was adjusted to 7 using 1 M NaOH, and the final volume was brought to 20 mL with distilled water. The samples were then incubated in a shaker incubator (MCİ55; Microtest, Turkey) at 37°C for 2 h.

After intestinal digestion, the samples from both gastric and intestinal digestion were centrifuged (3‐18K; Sigma) at 10,000 *g* for 10 min, and the supernatants were collected for analysis.

### 2.8 Total Phenolic Content and Antioxidant Capacity

Total phenolic content of the samples was determined by the Folin–Ciocalteu method. A 10‐g sample was centrifuged (3‐18K; Sigma) at 12,000 rpm for 15 min. One milliliter of the supernatant was separated, followed by 1 mL of 95% ethanol, 5 mL of distilled water, and 0.5 mL of 50% Folin solution. The resulting mixture was incubated in the dark for 5 min. Then, 5% Na_2_CO_3_ was added to the mixture and the solution was kept in the dark for 1 h. Absorbance values were recorded at a wavelength of 725 nm using a spectrophotometer (Lambda 25 UV/Vis; PerkinElmer, Singapore). The phenolic content of the samples was determined as gallic acid equivalents using a calibration curve (Apostolidis et al. 2006).

Antioxidant capacity of samples was determined using the 1,1‐Diphenyl‐2‐Picrylhydrazyl (DPPH) method. A 10 g sample of yogurt was centrifuged (3‐18K; Sigma) at 12,000 rpm for 15 min and subsequently filtered using Whatman No. 40 filter paper. Two milliliters of DPPH reagent (0.1 mM) prepared in methanol was combined with 100 μL of extract. Following a 30 min incubation in the dark, the absorbance was measured at 517 nm using a UV–Vis spectrophotometer (Lambda 25 UV/Vis; PerkinElmer, Singapore). The calculation of antioxidant capacity was conducted as follows.

$$\%\text{Antioxidant capacity}=\left(A_{c}-A_{s}\right)/A_{c}\times 100$$

where *A*_c_ = absorbance of the control sample and *A*_s_ = absorbance of the test sample.

Phenolic content and antioxidant analysis were performed on undigested samples and samples collected at different stages of digestion.

### 2.9 Bacterial Viability After Simulated In Vitro Digestion

After gastric and intestinal digestion, the digested samples were diluted decimal in Ringer's solution and incubated in the same conditions as before digestion on M17, MRS, and MRS‐sorbitol agar media for the enumeration of *S. thermophilus* , *Lb. bulgaricus*, and *L. acidophilus* , respectively.

### 2.10 Antimicrobial Activity

The disk diffusion method was used to determine the antimicrobial activity of the samples against *E. coli* (ATCC 25922). 10^6^ cfu/mL cell suspensions were grown in BHI (brain heart infusion) (Merck, Germany) medium. Nutrient agar was used as a medium to determine antimicrobial activity. Previously prepared nutrient agar was poured into petri dishes. *E. coli* was inoculated into petri dishes with a sterile swap. Blank paper disks were placed in the inoculated petri dishes. Yogurt samples were centrifuged (3‐18K; Sigma) at 10,000 rpm for 10 min and 20 μL of the supernatant obtained from each sample was taken and pipetted onto blank paper disks (Bioanalyse, Turkey). Ofloxacin (5 μq; Bioanalyse, Turkey) disk was placed in each petri dish as a positive control. Zone diameters were measured at the end of 24 h of incubation at 35°C (Bauer et al. 1966). Antimicrobial activity was applied to undigested samples and samples collected at different stages of digestion.

### 2.11 Color Analysis and Water Holding Capacity

A colorimeter (Ser‐Lab SL400, Turkiye) was used for color analysis. Measurements were made using the CIE color space, which includes L* (lightness), a* (red‐green), and b* (yellow‐blue) values. To measure the color differences between the control sample and others, the total color difference (∆*E*) was calculated using the formula given below.

$$\text{∆}E=\surd \left[\;\left(L2*-L1*\right)\;2+\left(a2*-a1*\right)\;2+\left(b2*-b1*\right)\;2\right]$$

The water holding capacity (WHC) of the samples was determined according to Isanga and Zhang (2009). The results were calculated as follows:

$$\mathrm{WHC}\;\left(\%\right)=\left(1-W_{1}/W_{2}\right)\times 100$$

*W*_1_: weight of whey after centrifugation, *W*_2_: sample weight.

### 2.12 Textural Properties

Textural properties of yogurts were determined using the texture profile analyzer (TX.TA2 Stable Micro Systems, Godalming, UK). The samples were subjected to back extrusion testing with a 5 kg cell load and a 35 mm probe were used for this process. The distance and test speed were 15 mm and 1 mm/s, respectively. Firmness and consistency values were obtained. All measurements were made at 5°C.

### 2.13 Sensory Evaluation

Sensory evaluation of the yogurt samples was conducted by seven panelists who regularly consumed milk and dairy products. The panelists, aged 35–55 years, included five women and two men, all members of the Dairy Technology Department with prior training in sensory evaluation of dairy products. Sensory evaluation was performed on Days 1, 11, and 21 according to the scoring card described by Meilgaard et al. (2006). Panelists evaluated samples for appearance, texture, taste, and overall acceptability using a 9‐point hedonic scale (1: Dislike extremely, 2: Dislike very much, 3: Dislike moderately, 4: Dislike slightly, 5: Neither like nor dislike, 6: Like slightly, 7: Like moderately, 8: Like very much, 9: Like extremely). For sensory evaluation, samples were randomly coded with 3‐digit numbers, placed in plastic containers, and presented to the panelists with a glass of water to improve mouth taste.

### 2.14 Statistical Analysis

All analyses were performed in duplicate. Randomized block design analysis of variance method was used in the statistical analysis of the obtained data. Using the Minitab package program (version 19; Minitab Inc., State College, PA), statistical analyses of the data were performed. Tukey's Multiple Range Test was applied with significance levels of *p* < 0.05.

## 3 Results and Discussion

### 3.1 Gross Composition and pH

**FIGURE 2:**
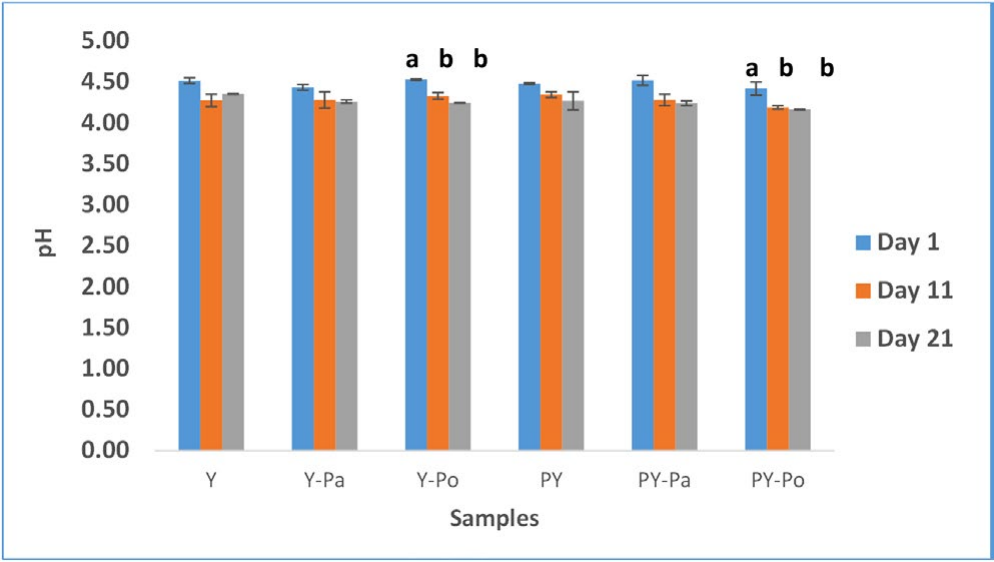
pH changes of samples. Different lowercase letters in the graph indicate differences between storage days (*p* < 0.05). PY, non‐fortified probiotic yogurt; PY‐Pa, probiotic yogurt fortified with 10^9^ heat‐killed *Lactobacillus acidophilus* cell; PY‐Po, probiotic yogurt fortified with autolyzed and dried *Saccharomyces cerevisiae*; Y, non‐fortified yogurt (control); Y‐Pa, yogurt fortified with heat‐killed *L. acidophilus* cell; Y‐Po, yogurt fortified with autolyzed and dried *S. cerevisiae*.

The composition of yogurt samples fortified with paraprobiotics and postbiotics and the pH change during 21 days of cold storage are given in Table 2 and Figure 2, respectively. The research findings indicated that adding paraprobiotic/postbiotic yogurt or probiotic yogurt did not significantly affect the compositional properties of the samples except for total protein (*p* > 0.05). However, a significant increase in total solid and protein content was observed in samples enriched with autolyzed *S. cerevisiae* (*p* < 0.05). This increase in protein is probably due to the protein structure of yeast cells (Takalloo et al. 2020). In contrast, the proportional amounts of protein and total solids in paraprobiotic‐added samples were found to be slightly lower than in the other samples.

**TABLE 2:** Composition of yogurt samples on Day 1 (mean ± SE).

	Fat (%)	Protein (%)	Dry matter (%)
Y	3.50 ± 0.05	4.82 ± 0.04^b^	16.97 ± 0.02
Y‐Pa	3.62 ± 0.21	4.79 ± 0.02^b^	16.79 ± 0.01
Y‐Po	3.55 ± 0.06	4.95 ± 0.01^a^	17.08 ± 0.01
PY	3.60 ± 0.56	4.90 ± 0.01^b^	16.96 ± 0.02
PY‐Pa	3.60 ± 0.23	4.80 ± 0.02^b^	16.92 ± 0.04
PY‐Po	3.55 ± 0.03	4.94 ± 0.01^a^	17.26 ± 0.00

*Note:* Different lowercase letters in the same column indicate differences between samples (*p* < 0.05). Non‐lettering columns indicate that the differences are not significant (*p* > 0.05).

Abbreviations: PY, non‐fortified probiotic yogurt; PY‐Pa, probiotic yogurt fortified with 10^9^ heat‐killed *L. acidophilus* cell; PY‐Po, probiotic yogurt fortified with autolyzed and dried *S. cerevisiae* ; Y, non‐fortified yogurt (control); Y‐Pa, yogurt fortified with heat‐killed *L. acidophilus* cell; Y‐Po, yogurt fortified with autolyzed and dried *S. cerevisiae* .

The initial pH values of the control and fortified yogurt samples ranged from 4.52 to 4.48. The pH value decreased slightly during the 21‐day storage period, and the lowest pH value measured at the end of storage was 4.17 for the PY‐Po sample. A decrease in pH values was observed in all samples as storage progressed. During storage, the LAB present break down lactose into lactic acid, resulting in a decrease in pH (Alizadeh Behbahani et al. 2023). The decreases in only the yogurt samples (P‐Po and PY‐Po) added with postbiotics during storage were found to be significant (*p* < 0.05). The decrease in pH in these samples containing autolyzed yeast during storage may be due to the use of β‐glucan in the yeast cell wall by lactic acid bacteria as food, thus increasing their ability to produce acid. Similar results were reported by Al‐Manhel and Niamah (2017) who investigated the use of mannan extract obtained from *S. cerevisiae* in producing probiotic yogurt.

### 3.2 Viability of Probiotics and Yogurt Bacteria

Several factors such as pH, product matrix and formulation, microbial interactions, the presence of additives, hydrogen peroxide and dissolved oxygen levels, concentration of acidic metabolites, and the buffering capacity of the product during shelf life affect bacterial survival in yogurt (Karimi et al. 2011; Mousavi et al. 2019). Change in the viable counts of yogurt and probiotic bacteria are given in Table 3. There was no significant difference in *S. thermophilus* in yogurt samples (*p* > 0.05). Mani‐López et al. (2014) reported that the number of *S. thermophilus* in probiotic yogurt was not affected by the presence of *L. acidophilus* . In addition, many studies have stated that the viability of *S. thermophilus* is more stable than other bacteria (Michael et al. 2015; Turgut and Cakmakci 2018). There were differences in the number of *Lb. bulgaricus* among the samples during cold storage (*p* < 0.05). However, this difference was independent of the addition of paraprobiotic or postbiotic. All probiotic yogurt applications, such as PY, PY‐Pa, and PY‐Po, significantly increased the number of *Lb. bulgaricus* compared to other yogurts. Acidophilin LA‐1, a bacteriosin synthesized by *L. acidophilus* during fermentation, inhibits the growth of *Lb. bulgaricus* (Özer 2006). In addition, the competition between probiotic and yogurt bacteria may have caused the lower number of *Lb. bulgaricus* in the production of probiotic dairy products. On the 21st day of storage, the *Lb. bulgaricus* count in the Y‐Po sample was lower than all other samples. Among the probiotic yogurt samples, the *Lb. bulgaricus* count in the PY‐Po sample was also lower. This result may be due to the fact that acidity is a critical factor affecting the viability of *Lb. bulgaricus*. These samples had the lowest pH value at the end of storage. The extreme decrease in pH may be related to the low lactobacilli count in these samples.

**TABLE 3:** Viability of yogurt and probiotic bacteria (log/cfu g).

	Samples	Period of storage		
		Day 1	Day 11	Day 21
*S. thermophilus*	Y	8.42 ± 0.35	8.84 ± 0.16	8.81 ± 0.10
	Y‐Pa	8.45 ± 0.41	8.86 ± 0.08	8.84 ± 0.07
	Y‐Po	8.44 ± 0.45	8.91 ± 0.05	8.86 ± 0.03
	PY	8.47 ± 0.38	8.87 ± 0.11	8.16 ± 0.55
	PY‐Pa	8.39 ± 0.39	8.83 ± 0.01	8.52 ± 0.09
	PY‐Po	8.62 ± 0.34	9.05 ± 0.10	8.94 ± 0.05
*L. delbrueckii* subsp. *bulgaricus*	Y	4.81 ± 0.13^b^	4.78 ± 0.06^b^	4.55 ± 0.31^c^
	Y‐Pa	4.57 ± 0.10^b^	4.54 ± 0.16^b^	3.95 ± 0.09^c^
	Y‐Po	4.57 ± 0.16^b^	4.48 ± 0.40^b^	3.83 ± 0.07^c^
	PY	7.78 ± 0.05^a^	7.80 ± 0.10^a^	7.72 ± 0.06^a^
	PY‐Pa	7.80 ± 0.02^aA^	7.78 ± 0.00^aA^	7.60 ± 0.01^aB^
	PY‐Po	7.82 ± 0.02^aA^	7.54 ± 0.24^aA^	6.02 ± 0.06^bB^
*L. acidophilus*	PY	7.78 ± 0.07	7.73 ± 0.08	7.67 ± 0.03^a^
	PY‐Pa	7.85 ± 0.02	7.57 ± 0.24	7.64 ± 0.04^a^
	PY‐Po	7.84 ± 0.08	6.83 ± 0.36	6.41 ± 0.29^b^

*Note:* Different lowercase letters in the same column indicate differences between samples (*p* < 0.05). Different uppercase letters in the same row indicate differences between storage days (*p* < 0.05). Non‐lettering columns and rows indicate that the differences are not significant (*p* > 0.05).

Abbreviations: PY, non‐fortified probiotic yogurt; PY‐Pa, probiotic yogurt fortified with 10^9^ heat‐killed *L. acidophilus* cell; PY‐Po, probiotic yogurt fortified with autolyzed and dried *S. cerevisiae* ; Y, non‐fortified yogurt (control); Y‐Pa, yogurt fortified with heat‐killed *L. acidophilus* cell; Y‐Po, yogurt fortified with autolyzed and dried *S. cerevisiae* .

The number of *L. acidophilus* in probiotic yogurts decreased slightly by Day 21. Acidic conditions in fermented products and competition between probiotic/non‐probiotic lactic acid bacteria result in a decrease in probiotic microorganisms (Vahdat et al. 2024). The effect of inactive *L. acidophilus* and autolyzed *S. cerevisiae* samples added to probiotic yogurts on the viability of *L. acidophilus* was insignificant (*p* > 0.05). Only the decrease in the *L. acidophilus* viable count of the PY‐Po sample at the end of storage was significant (*p* < 0.05). Many researchers have also reported a decrease in *L. acidophilus* counts in yogurt during cold storage, and this decrease has been associated with increased lactic acid and decreased nutrients during storage (Jooyandeh and Alizadeh Behbahani 2024; Jooyandeh et al. 2023). Although the number of probiotics gradually decreased during storage, it remained at the recommended level for fermented products to be considered as probiotic food.

### 3.3 Bacterial Viability After Simulated In Vitro Digestion

**FIGURE 3:**
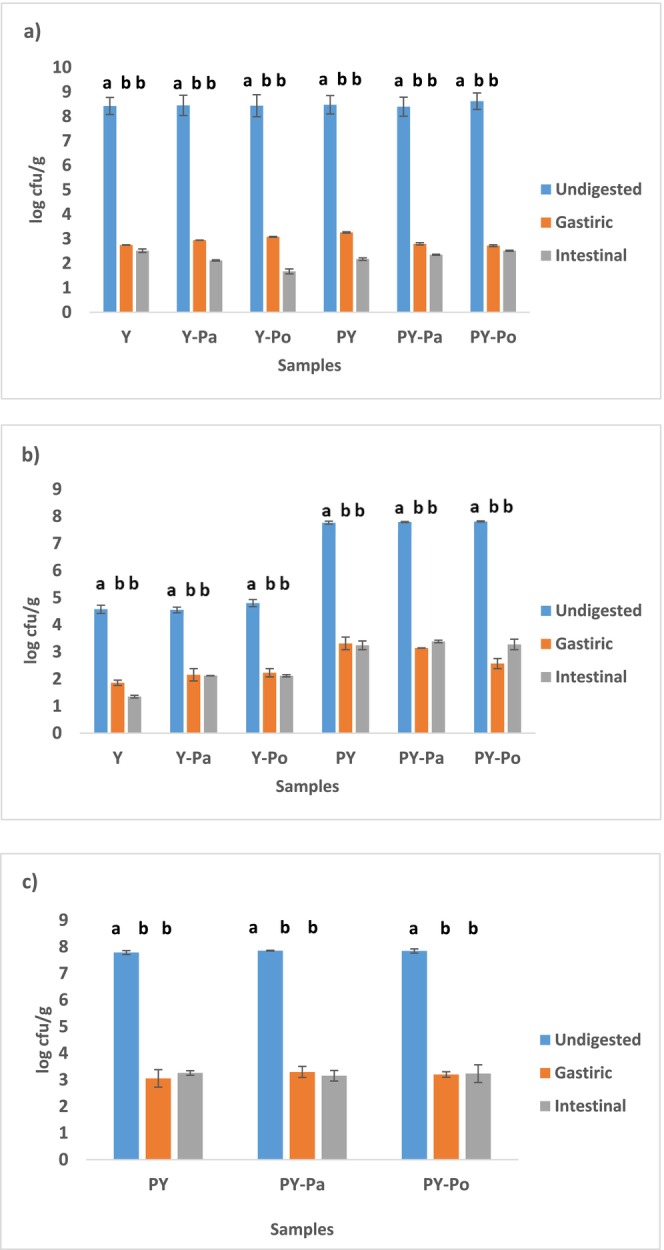
Bacterial survival following in vitro gastrointestinal digestion. (a) *Streptococcus thermophilus*, (b) *Lactobacillus delbrueckii bulgaricus*, and (c) *Lactobacillus acidophilus*. Different lowercase letters in the graph indicate differences between digestion phase (*p* < 0.05). PY, non‐fortified probiotic yogurt; PY‐Pa, probiotic yogurt fortified with 10^9^ heat‐killed *L. acidophilus* cell; PY‐Po, probiotic yogurt fortified with autolyzed and dried *Saccharomyces cerevisiae*; Y, non‐fortified yogurt (control); Y‐Pa, yogurt fortified with heat‐killed *L. acidophilus* cell; Y‐Po, yogurt fortified with autolyzed and dried *S. cerevisiae*.

Determining the viability of probiotics in gastrointestinal conditions is at least as important as assessing the viability of foods throughout their shelf life. Because for probiotics to have positive health effects, they must remain viable at certain levels throughout the gastrointestinal tract (Alizadeh Behbahani and Noshad 2024). Since yogurt bacteria cannot provide these conditions, they cannot show as beneficial health effects as probiotics. For the putative health benefits associated with probiotics to be achieved, a sufficient amount of viable cells must be present at the time of consumption (Tamime et al. 2006) Bacterial viability of yogurt samples after simulated gastrointestinal digestion on the first day of storage is presented in Figure 3. A significant decrease was observed in all bacterial species during gastric and intestinal digestion compared to before digestion (*p* < 0.05). A decrease of 5.90–5.21 log cfu/g was recorded in the number of *S. thermophilus* after the gastric digestion. In terms of *Lb. bulgaricus* count, a decrease of 2.71–2.41 log cfu/g was observed in plain yogurts at the gastric digestion, whereas a decrease of 5.25–4.47 log cfu/g was observed in probiotic yogurts. *L. acidophilus* count decreased by 4.73, 4.56, and 4.64 log cfu/g in PY, PY‐Pa and PY‐Po samples at the gastric stage, respectively. The highest decrease in viability in terms of *L. acidophilus* counts was observed during gastric digestion. These findings are consistent with previous studies reporting a decrease in *L. acidophilus* counts during simulated gastric digestion (Casarotti and Penna 2015; Gocer et al. 2021; Khorshidi et al. 2021; Ranadheera et al. 2012). At the gastric digestion, the PY‐Pa sample had the highest viability rate for all three bacterial species.

After intestinal digestion, a 6.3–6.7 log cfu/g decrease in the number of *S. thermophilus* was observed. Overall, *S. thermophilus* was more sensitive to the gastrointestinal environmental conditions than *Lb. bulgaricus*. It has been reported that *S. thermophilus* has low resistance to bile salts (Ziar et al. 2014). The number of *Lb. bulgaricus* decreased by 3–4 log cfu/g after intestinal digestion. The number of *L. acidophilus* decreased by 4.53, 4.50 and 4.61 log cfu/g in PY, PY‐Pa and PY‐Po samples, respectively. The decreased viability of *L. acidophilus* may be attributed to the high acidity of gastric juice and the antimicrobial effect of the pepsin enzyme (Mousanejadi et al. 2023). No difference was found in the number of *L. acidophilus* in probiotic yogurts after gastric and intestinal digestion (*p* > 0.05). The highest survival for each bacteria after intestinal digestion was in the PY‐Pa sample. Although the viability levels of all bacteria generally decreased after intestinal digestion compared to gastric digestion, the number of *Lb. bulgaricus* in PY‐Pa and PY‐Po samples showed a slight increase after intestinal digestion. The viable count of *L. acidophilus* in probiotic yogurt samples after exposure to digestive fluids was approximately 4 log cfu/g. This count is lower than the appropriate dose to provide probiotic benefits. Probiotic microorganisms must remain viable at a level of approximately 10^6^–10^9^ cfu/g or mL after processing, storage, and even digestion (Sarkar 2013).

Resistance to bile salts in the intestine is an important criterion for selecting probiotic bacteria (Alizadeh Behbahani and Noshad 2024). However, it is challenging to simulate exact in vivo conditions in vitro, and not all parameters that may affect bacterial viability are considered. The external conditions before entering the small intestine determine the effects of bile on a strain. Exposure to various pHs, temperatures, and growth atmospheres can make bacteria resistant or sensitive to the effects of bile. In the intestine, bacteria may not be exposed to bile due to the influence of the food matrix, or the food component may bind bile acids, which in turn affects bacterial viability (Begley et al. 2005).

### 3.4 Effect of In Vitro Digestion of Addition of Autolyzed *S. cerevisiae* and Inactive *L. acidophilus* on Total Phenolics and Antioxidant Activity of Yogurts

Changes in undigested and post‐digestion phenolic content and antioxidant capacity of yogurt samples are given in Table 4. For undigested samples, the phenolic content of the samples increased significantly compared to the control sample with the addition of postbiotics (*p* < 0.05). Control sample had the lowest TPC value and had a significantly lower phenolic content than all other samples (*p* < 0.05). The Folin–Ciocalteu reactivity of yogurt is attributed to the presence of milk compounds apart from polyphenols such as free amino acids, low molecular weight antioxidants, proteins, and peptides (Helal and Tagliazucchi 2018). Paraprobiotic‐added Y‐Pa sample slightly increased the TPC level compared to the control. However, the TPC value of the Y‐Po sample fortified with postbiotics was found to be significantly higher than the control (*p* < 0.05). The TPC values of the paraprobiotic and postbiotic‐fortified probiotic yogurt samples (PY‐, PY‐Pa, and PY‐Po) were higher than those of the other samples. The PY‐Po sample, in particular, had the highest phenolic content among all samples (*p* < 0.05). PY and PY‐Pa samples also showed high phenolic content. These findings suggest that postbiotics are particularly effective in increasing phenolic compounds and may have a synergistic effect when applied to probiotic yogurt. Therefore, postbiotics may come to the fore in functional yogurt formulations developed to increase the content of biologically active compounds.

**TABLE 4:** Changes in the total phenolic content (TPC) and total antioxidant capacity (TAC) of yogurt during in vitro gastrointestinal digestion.

	Undigested	Gastric digestion	Intestinal digestion
TPC (mg GAE/100 g)			
Y	41.24 ± 0.10^dC^	51.83 ± 0.15^bB^	112.37 ± 5.13 ^A^
Y‐Pa	43.10 ± 0.14^cdC^	54.23 ± 0.37^abB^	114.84 ± 2.37^A^
Y‐Po	48.83 ± 0.35^abC^	53.41 ± 0.17^bB^	115.01 ± 1.25^A^
PY	46.65 ± 0.48^abC^	53.29 ± 0.66^bB^	106.12 ± 3.91^A^
PY‐Pa	45.90 ± 1.01^bcC^	56.96 ± 1.01^aB^	110.98 ± 3.30^A^
PY‐Po	49.54 ± 0.45^aC^	53.47 ± 0.18^bB^	116.60 ± 3.25^A^
TAC (%)			
Y	6.88 ± 0.11^bC^	12.33 ± 0.22^cB^	21.04 ± 0.37^A^
Y‐Pa	6.65 ± 0.46^bC^	13.81 ± 0.25^bcB^	20.42 ± 0.99^A^
Y‐Po	7.84 ± 0.56^abC^	15.06 ± 0.07^abB^	21.11 ± 0.62^A^
PY	7.80 ± 0.35^abC^	14.19 ± 0.76^abcB^	20.77 ± 0.70^A^
PY‐Pa	6.78 ± 0.02^bC^	14.73 ± 0.02^abB^	20.93 ± 0.32^A^
PY‐Po	9.42 ± 0.63^aC^	15.96 ± 0.04^aB^	21.00 ± 0.57^A^

*Note:* Different capital letters in rows or different lower case letters in columns indicate statistically significant differences (*p* < 0.05).

Abbreviations: PY, non‐fortified probiotic yogurt; PY‐Pa, probiotic yogurt fortified with 10^9^ heat‐killed *L. acidophilus* cell; PY‐Po, probiotic yogurt fortified with autolyzed and dried *S. cerevisiae* ; Y, non‐fortified yogurt (control); Y‐Pa, yogurt fortified with heat‐killed *L. acidophilus* cell; Y‐Po, yogurt fortified with autolyzed and dried *S. cerevisiae* .

Yogurt bacteria produce various bioactive peptides during fermentation, which increases the antioxidant activity of yogurt. The antioxidant activity of yogurt can be further increased by using bioactive plant extracts and probiotic microorganisms (Sıçramaz 2025). The TAC values of the yogurt samples showed significant differences depending on both the addition of probiotic bacteria (Y or PY) and the addition of paraprobiotics or postbiotics (*p* < 0.05). Among all samples, the PY‐Po sample had higher TAC value than all other samples (*p* < 0.05). This result suggests that the combination of probiotics and postbiotics has a synergistic effect on enhancing the antioxidant potential of yogurt, likely due to the increased presence of bioactive compounds and fermentation‐derived metabolites. It has been reported that probiotic bacteria produce higher amounts of bioactive peptides and have higher antioxidant activities than yogurt bacteria (Alizadeh Behbahani and Noshad 2024). This may explain why probiotic yogurt shows higher antioxidant activity than plain yogurt (Liu et al. 2024; Sah et al. 2014). Y‐Po and PY had TAC values of 7.84% ± 0.56% and 7.80% ± 0.35%, respectively. This suggests that both the addition of postbiotics alone and the use of probiotic cultures contributed significantly to the antioxidant activity, although not as strongly as when used in combination. 80%–90% of the cell wall of *Saccharomyces* ssp. consists of polysaccharides, mannans, and glucans, and these cell mannans have been reported to have antioxidant, antibacterial, and antimutagenic properties (Liu et al. 2021). In contrast, samples containing paraprobiotics (Y‐Pa and PY‐Pa) and the control sample (Y) showed the lowest TAC values (between 6.65% ± 0.46% and 6.88% ± 0.11%). This suggests that heat‐inactivated cells may have limited efficacy in increasing antioxidant capacity compared to live probiotics or postbiotics. Overall, the inclusion of postbiotics—especially in combination with probiotics—significantly increased the antioxidant capacity of yogurt. These findings support the functional potential of postbiotic‐fortified yogurt products to provide health benefits beyond basic nutrition.

In order for bioactive compounds to have a positive effect on health, they must be released from the food matrix and remain stable in gastrointestinal conditions (Helal and Tagliazucchi 2018). Therefore, it is critical to evaluate the bioactivity of fortified foods after the digestive process. A statistically significant increase (*p* < 0.05) was observed in both TPC and TAC values from the undigested phase to the intestinal phase throughout the digestion process (Table 4). After gastric digestion, significant increases were recorded in the TPC values of all samples compared to the TPC values of undigested samples (*p* < 0.05). After gastric digestion, TPC of the samples increased by 8%–25% compared to before digestion, whereas this rate increased further after intestinal digestion, reaching 135%–172%. This increase suggests that the gastrointestinal environment facilitates the release of bound phenolic compounds from the food matrix, especially in the intestinal phase. Hydrolysis of caseins occurring in the intestinal phase released bound compounds, resulting in an increase in phenolic compounds (Helal and Tagliazucchi 2018). A significant increase in TPC values was observed in all samples after intestinal digestion compared to pre‐digestion and gastric digestion (*p* < 0.05). Data indicate that the use of postbiotics alone or in combination with probiotics significantly increases the release and bioaccessibility of phenolic compounds. Similar to TPC, TAC values increased significantly with digestion in all samples (*p* < 0.05). Among the undigested samples, TAC values ranged from 6.65% to 9.42%. The TAC value of the PY‐Po sample was significantly higher than that of the other samples (*p* < 0.05). After gastric digestion, the antioxidant capacity of the samples increased by 69%–117%, whereas after intestinal digestion, the antioxidant capacity increased by 122%–208% compared to before digestion. After gastric digestion, the TAC value of the PY‐Po sample was found to be significantly higher than the other samples compared to before digestion (*p* < 0.05). This can be attributed to the release of antioxidant substances by the pepsin enzyme, which breaks down yeast cells and inactive cells during gastric digestion. In addition, hydrolysis of milk proteins during gastrointestinal digestion may release bioactive peptides that increase antioxidant activity (Kashung and Karuthapandian 2025). TAC values of all samples after intestinal digestion were higher than after gastric digestion. Neutral pH in the intestinal phase after gastric digestion may have contributed positively to increased antioxidant activity (Caponio et al. 2022). However, in the intestinal phase, TAC values among all samples exhibited closer values (approximately 20%–21%) and no statistically significant difference was detected at this stage (*p* > 0.05). The findings indicate that gastrointestinal digestion significantly increased the bioaccessibility of both phenolic compounds and antioxidant capacity in yogurt formulations. In particular, the inclusion of postbiotics in yogurt, alone or together with probiotics, significantly improved these functional properties. These results support the potential use of postbiotic‐fortified yogurts as effective functional foods to improve antioxidant uptake and bioavailability in the human diet.

### 3.5 Antimicrobial Activity

The antimicrobial activity of yogurt samples, fortified with paraprobiotic, and postbiotic components, was evaluated through the inhibition zone diameters (mm) against *E. coli* (ATCC 25922) at different stages of digestion (Table 5). Ofloxacin was used as a positive control and demonstrated the maximum inhibition across all digestion phases. Among the undigested samples, the PY‐Po samples (9.0 ± 0.0 mm) exhibited the highest antimicrobial activity, which was significantly different (*p* < 0.05) from the control yogurt (7.0 ± 0.0 mm). This result suggests that postbiotic‐fortified probiotic yogurt has a significantly higher antimicrobial potential even before digestion. The antimicrobial activity in this sample may be due to the cell wall mannans of autolyzed *S. cerevisiae* and the bioactive components released by fermentation of probiotic microorganisms. Y and Y‐Pa exhibited the lowest antimicrobial activity. Addition of inactive *L. acidophilus* cells to yogurt or probiotic yogurt had no significant effect on antimicrobial activity (*p* > 0.05). Antimicrobial metabolites are produced by lactic acid bacteria during yogurt fermentation. These metabolites produced have a significant inhibitory effect on the growth of pathogenic microorganisms. In addition, low temperature storage (+4°C) and high acidity are also factors that limit the growth of pathogenic bacteria (Azizkhania et al. 2020). Among lactic acid bacteria, *L. acidophilus* is a probiotic that produces bacteriocins with broad‐spectrum antimicrobial activity (Menezes et al. 2023). In addition, it has been reported that antimicrobial activity occurs when probiotic microorganisms produce metabolites such as organic acids, diacetyl, hydrogen peroxide, ethanol, carbon dioxide, acetoin, acetaldehyde, reutericyclines, reuterins and bacteriocins (Zibaei‐Rad et al. 2023). This may explain the relatively higher antimicrobial activity observed in probiotic yogurts.

**TABLE 5:** Antimicrobial activity of yogurt samples (mm inhibition zone diameter).

	Undigested	Gastric digestion	Intestinal digestion
Ofloxacin	16.0 ± 0.0^a^	16.0 ± 0.0^a^	16.0 ± 0.0^a^
Y	7.0 ± 0.0^c^	8.0 ± 0.0^b^	7.5 ± 0.5^b^
Y‐Pa	7.5 ± 0.5^c^	8.0 ± 0.0^b^	7.5 ± 0.25^b^
Y‐Po	8.0 ± 0.0^bc^	8.5 ± 0.5^b^	8.0 ± 0.0^b^
PY	8.0 ± 0.0^bc^	8.5 ± 0.5^b^	8.5 ± 0.0^b^
PY‐Pa	8.0 ± 0.0^bc^	8.5 ± 0.5^b^	8.5 ± 0.0^b^
PY‐Po	9.0 ± 0.0^b^	9.5 ± 0.5^b^	9.0 ± 0.0^b^

*Note:* Different lower‐case letters in columns indicate statistically significant differences (*p* < 0.05).

Abbreviations: PY, non‐fortified probiotic yogurt; PY‐Pa, probiotic yogurt fortified with 10^9^ heat‐killed *L. acidophilus* cell; PY‐Po, probiotic yogurt fortified with autolyzed and dried *S. cerevisiae* ; Y, non‐fortified yogurt (control); Y‐Pa, yogurt fortified with heat‐killed *L. acidophilus* cell; Y‐Po, yogurt fortified with autolyzed and dried *S. cerevisiae* .

During gastric digestion, a slight increase in antimicrobial activity was observed across all samples, potentially due to the low pH of gastric digestion, matrix breakdown and the presence of bioactive peptides released after yogurt digestion. However, no statistically significant differences were found among fortified samples (*p* > 0.05). After simulated intestinal digestion, the antimicrobial activities remained relatively stable. Although the PY‐Po sample had the highest inhibition zone diameter, the Y and Y‐Pa samples had the lowest zone diameter. The other fortified samples also maintained modest but consistent activity (8.0–8.5 mm). Although previous studies reported that *L. acidophilus* has a strong antimicrobial effect on pathogenic bacteria (Soltani et al. 2022; Tahmasebi and Mofid 2021), the current study found weaker antimicrobial activity. The antimicrobial activity of probiotics varies significantly across species and strains (Chornchoem et al. 2025). The findings indicated that probiotic yogurts had a bacteriostatic effect against *E. coli* , and this effect was slightly enhanced by the addition of a postbiotic.

### 3.6 Textural and Color Properties

The textural properties, WHC and color parameters of yogurt samples measured on the 1st, 11th and 21st days of storage are given in Table 6. It was determined that all samples maintained their initial firmness values throughout the storage period (*p* > 0.05). It was observed that the addition of postbiotics/paraprobiotics to yogurts did not have a statistically significant effect on the hardness values during the storage period (*p* > 0.05). Similarly, although the consistency values increased and decreased during the storage period, these fluctuations were not statistically significant (*p* > 0.05).

**TABLE 6:** Textural and color properties of samples.

	Samples	Period of storage		
		Day 1	Day 11	Day 21
Firmness (g)	Y	567.47 ± 24.09	550.79 ± 22.67	646.49 ± 11.53
	Y‐Pa	513.73 ± 14.22	580.52 ± 40.22	589.67 ± 15.91
	Y‐Po	500.17 ± 75.22	533.98 ± 59.17	539.50 ± 13.33
	PY	474.91 ± 45.64	550.06 ± 27.76	598.115 ± 12.31
	PY‐Pa	479.38 ± 50.08	530.65 ± 15.28	560.70 ± 7.47
	PY‐Po	594.80 ± 7.84	624.535 ± 51.86	649.53 ± 82.75
Consistency (g.s)	Y	6808.28 ± 294.60	6055.27 ± 564.29	6958.99 ± 236.11
	Y‐Pa	6070.08 ± 190.53	6603.12 ± 551.04	6829.92 ± 117.80
	Y‐Po	5358.48 ± 313.13	6572.91 ± 219.49	6504.53 ± 14.29
	PY	5458.99 ± 350.75	6661.85 ± 496.86	7304.99 ± 37.85
	PY‐Pa	5669.38 ± 485.65	6448.96 ± 243.93	6681.34 ± 172.58
	PY‐Po	6801.32 ± 22.84	7433.785 ± 61.93	7729.13 ± 788.05
WHC (%)	Y	35.92 ± 2.56	32.71 ± 0.71	35.01 ± 1.31
	Y‐Pa	36.65 ± 1.08	33.89 ± 0.33	35.17 ± 1.18
	Y‐Po	37.32 ± 1.54	33.90 ± 0.99	34.94 ± 2.22
	PY	36.46 ± 1.93	34.80 ± 0.59	35.20 ± 2.37
	PY‐Pa	37.77 ± 1.16	34.40 ± 0.12	35.49 ± 1.70
	PY‐Po	38.31 ± 1.32	35.15 ± 2.01	35.89 ± 2.50
*L*	Y	83.05 ± 0.05^a^	82.75 ± 0.15^a^	83.00 ± 0.00^a^
	Y‐Pa	82.95 ± 0.05^a^	82.70 ± 0.10^a^	83.05 ± 0.05^a^
	Y‐Po	82.00 ± 0.1^cAB^	81.65 ± 0.05^bB^	82.25 ± 0.05^bA^
	PY	83.00 ± 0.2^a^	82.35 ± 0.15^a^	83.05 ± 0.05^a^
	PY‐Pa	82.9 ± 0.1^ab^	82.70 ± 0.00^a^	82.95 ± 0.05^a^
	PY‐Po	82.20 ± 0.2^bc^	81.60 ± 0.00^b^	82.25 ± 0.05^b^
a*	Y	0.85 ± 0.05^a^	1.05 ± 0.05^a^	0.95 ± 0.05^a^
	Y‐Pa	0.90 ± 0.00^a^	1.05 ± 0.05^a^	0.90 ± 0.00^a^
	Y‐Po	0.45 ± 0.05^b^	0.55 ± 0.05^b^	0.55 ± 0.05^b^
	PY	0.95 ± 0.05^aAB^	1.10 ± 0.00^aA^	0.85 ± 0.05^aB^
	PY‐Pa	0.95 ± 0.05^a^	1.10 ± 0.00^a^	1.05 ± 0.05^a^
	PY‐Po	0.45 ± 0.05^b^	0.55 ± 0.05^b^	0.50 ± 0.00^b^
b*	Y	7.65 ± 0.15^bc^	7.65 ± 0.05^b^	7.95 ± 0.05^b^
	Y‐Pa	7.7 ± 0.10^abc^	7.65 ± 0.05^b^	7.95 ± 0.00^b^
	Y‐Po	8.15 ± 0.05^aAB^	8.1 ± 0.00^aB^	8.3 ± 0.05^aA^
	PY	7.55 ± 0.05^c^	7.5 ± 0.00^b^	7.45 ± 0.00^c^
	PY‐Pa	7.6 ± 0.00^bcB^	7.65 ± 0.05^bAB^	7.8 ± 0.05^bA^
	PY‐Po	8.05 ± 0.05^ab^	8.1 ± 0.00^a^	8.25 ± 0.05^a^
Δ*E*	Y‐Pa	0.14 ± 0.00^b^	0.12 ± 0.02^b^	0.14 ± 0.04^b^
	Y‐Po	1.23 ± 0.00^a^	1.30 ± 0.11^a^	0.92 ± 0.02^a^
	PY	0.22 ± 0.12^b^	0.44 ± 0.30^b^	0.51 ± 0.10^b^
	PY‐Pa	0.24 ± 0.10^b^	0.16 ± 0.06^b^	0.19 ± 0.05^b^
	PY‐Po	1.04 ± 0.05^a^	1.34 ± 0.07^a^	0.93 ± 0.10^a^

*Note:* Different capital letters in rows or different lower case letters in columns indicate statistically significant differences (*p* < 0.05).

Abbreviations: PY, non‐fortified probiotic yogurt; PY‐Pa, probiotic yogurt fortified with 10^9^ heat‐killed *L. acidophilus* cell; PY‐Po, probiotic yogurt fortified with autolyzed and dried *S. cerevisiae* ; Y, non‐fortified yogurt (control); Y‐Pa, yogurt fortified with heat‐killed *L. acidophilus* cell; Y‐Po, yogurt fortified with autolyzed and dried *S. cerevisiae* .

Water holding capacity (WHC) is one of the important parameters in determining yogurt quality and is inversely proportional to syneresis. High WHC values result in low water release (Diamantino et al. 2014). Serum separation is observed in yogurts due to insufficient dry matter increase, rapid or slow acidity development during fermentation, or inadequate cooling after fermentation. In general, the WHC values of the samples showed slight changes during storage. The WHC value of the PY‐Po sample was slightly higher than that of the other samples at all storage days. This effect is likely due to the structural contributions of postbiotic components such as cell wall fragments and metabolites and the exopolysaccharide production ability of probiotic microorganisms. Similar results were also reported by Raikos et al. (2018). This may contribute positively to the texture and syneresis stability of the yogurt during shelf life.

The L* value, which is considered the most important determinant of the perceived appearance of yogurt, represents lightness (100) and blackness (0). a* values represent red/green (positive/negative) hues, whereas b* values represent yellow/blue (positive/negative) hues. The color properties of the yogurt samples, including L* (lightness), a* (red‐green axis), b* (yellow‐blue axis), and total color difference Δ*E*, were monitored over the storage period. These parameters are crucial as they influence consumer perception and product appeal.

All yogurt samples generally exhibited high L* values, indicating a bright, light‐colored product. Samples fortified with postbiotics (Y‐Po and PY‐Po) showed significantly lower L* values (*p* < 0.05), indicating darker appearance. This may be attributed to pigments originating from the postbiotic material. The a* values for the Y, Y‐Pa, PY and PY‐Pa samples remained within a narrow and positive range, indicating a slight red hue. In contrast, the postbiotic‐added samples (Y‐Po and PY‐Po) showed significantly lower a* values (*p* < 0.05), ranging from 0.45 to 0.55. In the PY sample, a significant decrease in a values was observed on the 21st day (*p* < 0.05). The b* value in the samples with added postbiotics (Y‐Po and PY‐Po) was found to be significantly higher than the other samples (*p* < 0.05). This increase in yellowness may be associated with the metabolic byproducts or natural pigments in postbiotics. On the other hand, the PY samples exhibited significantly lower b* values (*p* < 0.05), especially by Day 21, indicating a less yellow appearance compared to postbiotic‐fortified samples. Δ*E* values indicate the overall perceptible color difference from the control (sample Y). The highest Δ*E* values were observed in postbiotic samples (Y‐Po and PY‐Po), especially on Day 11 (*p* < 0.05). In contrast, Y‐Pa, PY, and PY‐Pa exhibited Δ*E* values below 0.5 throughout storage, indicating slight visual differences. Perceptible differences in color are classified as very distinct (Δ*E* > 3), distinct (1.5 < Δ*E* < 3), and slightly different (1.5 < Δ*E*). Therefore, the difference between samples is not perceptible because the ΔE value is less than 1.5. These results suggest that postbiotic addition significantly affects color characteristics, whereas paraprobiotic inclusion had minimal impact on color stability.

### 3.7 Sensorial Properties

**FIGURE 4:**
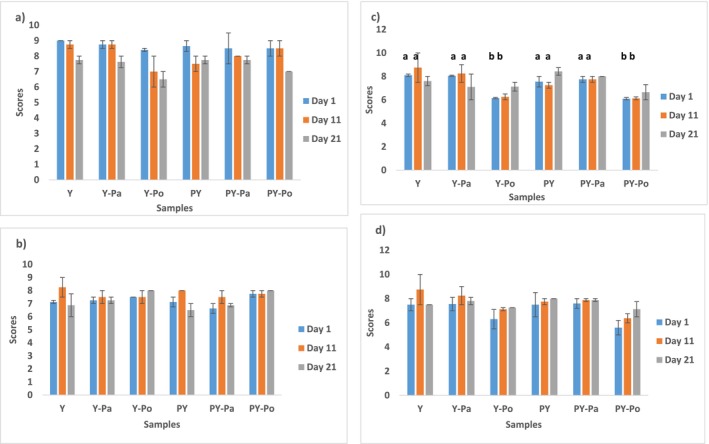
Sensory evaluation results of samples. (a) Appearance, (b) body, (c) taste, and (d) overall acceptability. Different lowercase letters in the graph indicate differences between storage days (*p* < 0.05). PY, non‐fortified probiotic yogurt; PY‐Pa, probiotic yogurt fortified with 10^9^ heat‐killed *Lactobacillus acidophilus* cell; PY‐Po, probiotic yogurt fortified with autolyzed and dried *Saccharomyces cerevisiae*; Y, non‐fortified yogurt (control); Y‐Pa, yogurt fortified with heat‐killed *L. acidophilus* cell; Y‐Po, yogurt fortified with autolyzed and dried *S. cerevisiae*.

Sensory evaluation plays a crucial role in enhancing product quality and extending shelf life. When new methods are developed to improve product composition, sensory evaluation becomes key to determining whether the new product will be acceptable to consumers. From the consumer's perspective, sensory attributes are the most direct criterion for product acceptance (Ruiz‐Capillas and Herrero 2021). If the new product does not taste favorable, it will not be commercially available. The sensory attributes of yogurt samples—appearance, body, taste, and overall acceptability—were evaluated on Days 1, 11, and 21 of storage and presented in Figure 4. The effect of adding inactive *L. acidophilus* or autolyzed *S. cerevisiae* to yogurts on sensory scores of samples was found to be significant only in terms of taste (*p* < 0.05). Although the samples coded Y‐Pa, PY and PY‐Pa did not differ significantly from the control sample, the taste scores of samples containing *S. cerevisiae* were significantly lower on Days 1 and 11 of storage (*p* < 0.05). Panelists stated that “umami” or “bouillon” taste was perceived in these samples. However, on the last day of storage, taste scores of these samples with the postbiotic addition were the same as those for other samples. This was probably due to the increase in acidic taste with the progress of storage and the suppression of umami taste by the formed flavor components. Although the taste scores of samples with added autolyzed *S. cerevisiae* were lower, they remained within acceptable limits (above 6 points on a scale where “like slightly” indicates acceptability). Yeast extract has gained importance as a food flavoring agent, following monosodium glutamate, due to its association with barbecue and meat‐like flavors (Tao et al. 2023) and used as a flavor enhancer in ready‐made food compositions (Das et al. 2022). The high temperature applied in the preparation of yeast extract is a very important factor in the formation of the “umami” taste produced from amino acids and peptides (Alim et al. 2019). In addition to these, “yeast taste” in yeast extract also consists of butyric and propionic acid and can negatively affect sensory evaluation (Tao et al. 2023). These flavor components present in yeast extract may limit the use of yeast in dairy products.

## 4 Conclusion

This study evaluated the potential effects of fortifying yogurt with postbiotics and paraprobiotics in today's world, where consumer interest in probiotic products has increased. The study findings indicated that the addition of inactive *L. acidophilus* to the yogurt formulation before fermentation did not cause a significant change in the physicochemical, microbiological, antioxidant, and antibacterial properties of the products. However, the addition of autolyzed and dried *S. cerevisiae* increased the protein content of yogurts, changed their color characteristics, and positively affected the total phenolic content, antioxidant capacity, and antimicrobial activity levels.

In vitro analyses performed at pre‐digestion, gastric, and intestinal stages revealed that samples containing postbiotics and paraprobiotics had higher values in terms of bioavailable components. This situation shows that postbiotic and paraprobiotic supplementation may be an effective strategy in terms of increasing the functional properties of yogurt. In addition, the advantages of these biocomponents, such as not interacting negatively with food components, not affecting product shelf life, ease of processing, and contributing to consumer health, support their applicability in the production of functional dairy products.

Despite the promising results, literature on the behavior of paraprobiotics and postbiotics in dairy products and their effects on in vitro digestive processes is limited. Therefore, advanced clinical and human intervention studies are needed to confirm these findings and enable more comprehensive evaluations. In addition, investigating the stability, sensory effects, and long‐term health effects of postbiotics and paraprobiotics in various dairy product matrices will provide valuable insights into this area.

## Author Contributions

**Gökçe Eminoğlu:** conceptualization, writing – original draft, data curation, resources, methodology, formal analysis, investigation, writing – review and editing.

## Funding

The author has nothing to report.

## Conflicts of Interest

The author declares no conflicts of interest.

## Data Availability

The data that support the findings of this study are available from the corresponding author upon reasonable request.
